# Longitudinal and Long-Term Wastewater Surveillance for COVID-19: Infection Dynamics and Zoning of Urban Community

**DOI:** 10.3390/ijerph19052697

**Published:** 2022-02-25

**Authors:** Athmakuri Tharak, Harishankar Kopperi, Manupati Hemalatha, Uday Kiran, Gokulan C. G., Shivranjani Moharir, Rakesh K. Mishra, S. Venkata Mohan

**Affiliations:** 1 Bioengineering and Environmental Sciences Lab, Department of Energy and Environmental Engineering (DEEE), CSIR-Indian Institute of Chemical Technology (CSIR-IICT), Hyderabad 500007, India; tharakathmakuri527@gmail.com (A.T.); chanty525@gmail.com (H.K.); hemalathamanupati@yahoo.com (M.H.); 2 Academy of Scientific and Innovative Research (AcSIR), Ghaziabad 201002, India; uday@ccmb.res.in; 3 CSIR-Centre for Cellular and Molecular Biology (CSIR-CCMB), Hyderabad 500007, India; gokulan@ccmb.res.in (G.C.G.); shivranjani@ccmb.res.in (S.M.)

**Keywords:** longitudinal sampling, SARS-CoV-2, RNA copies, infection rate, zoning, community spread

## Abstract

Wastewater-based epidemiology (WBE) is emerging as a potential approach to study the infection dynamics of SARS-CoV-2 at a community level. Periodic sewage surveillance can act as an indicative tool to predict the early surge of pandemic within the community and understand the dynamics of infection and, thereby, facilitates for proper healthcare management. In this study, we performed a long-term epidemiological surveillance to assess the SARS-CoV-2 spread in domestic sewage over one year (July 2020 to August 2021) by adopting longitudinal sampling to represent a selected community (~2.5 lakhs population). Results indicated temporal dynamics in the viral load. A consistent amount of viral load was observed during the months from July 2020 to November 2020, suggesting a higher spread of the viral infection among the community, followed by a decrease in the subsequent two months (December 2020 and January 2021). A marginal increase was observed during February 2021, hinting at the onset of the second wave (from March 2021) that reached it speak in April 2021. Dynamics of the community infection rates were calculated based on the viral gene copies to assess the severity of COVID-19 spread. With the ability to predict the infection spread, longitudinal WBE studies also offer the prospect of zoning specific areas based on the infection rates. Zoning of the selected community based on the infection rates assists health management to plan and manage the infection in an effective way. WBE promotes clinical inspection with simultaneous disease detection and management, in addition to an advance warning signal to anticipate outbreaks, with respect to the slated community/zones, to tackle, prepare for and manage the pandemic.

## 1. Introduction

The diagnostic aids, equipment and medical facilities have phenomenally improved since the emergence of the SARS-CoV-2 pandemic, in addition to the surveillance of SARS-CoV-2 using clinical data. However, employing swab samples obtained from person to person during critical periods of the pandemic is a challenge to understand spread among communities [1,2,3]. Wastewater based epidemiological study (WBE) is being conceived as one of the viable protocols that can infer the dynamics and infection rates of SARS-CoV-2 and its state of severity among the community [4,5,6,7]. WBE data will assist in detecting viruses in a community prior to clinical recognition, allowing preventive measure and precautions to be dispersed among the community to resist the outbreak [8,9,10,11]. The design of the sampling protocol is a crucial factor to detect the COVID-19 genetic material in the wastewater, since SARS-CoV-2 able to infect the gastrointestinal tract (GI) in addition to the bronchial inflammation [12,13,14,15,16,17,18]. A load of viral material temporally alters sewage based on the time of defecation frequencies and sampling [19,20]. Diverse viral load shedding from the affected community, convergence of household wastewater and industrial effluents and time of sampling could affect the detection of the viral genome in the sewage; however, WBE provides a range of information to predict the dynamics of infection with the design of a sampling protocol [7,13,14,15,21,22,23,24,25]. The development of highly sensitive and specific RT-PCR tests to detect SARS-CoV-2 (the corona virus causing the COVID-19 pandemic) in sewage samples presents the potential to use wastewater sampling as a diagnostic test for SARS-CoV-2 in the community [21,22,23,24].

Different independent SARS-CoV-2 WBE studies have followed various sewage sampling and processing methods to detect the SARS-CoV-2 RNA in sewage [7,23,24,25]. The grab sampling method can be used in remote areas and even in poor sewage system conditions, which aids in improving surveillance [22]. These kinds of WBE methods will help to monitor surveillance in the area where proper hospitality infrastructure is lacking. A truly representative sample at the selected station and its load provide information about the spread and impact of the infection that helps to provide early warning signals [25,26,27,28]. The current study made an attempt to investigate the persistence and dynamics of SARS-CoV-2 in wastewater by conducting longitudinal sampling over a period of one year ((July 2020 to August 2021), excluding the rainfall event months, i.e., August 2020 and September 2020)) in a selected community representing ~2.5 lakhs of population. A total of eight sampling points were selected in the Tarnaka and Nacharam areas of Hyderabad covering both lateral and main drains. Both weekly (during peak infection period) and monthly monitoring was conducted to predict the dynamics of the virus overtime.

## 2. Materials and Methods

### 2.1. Study Community

**Figure 1:**
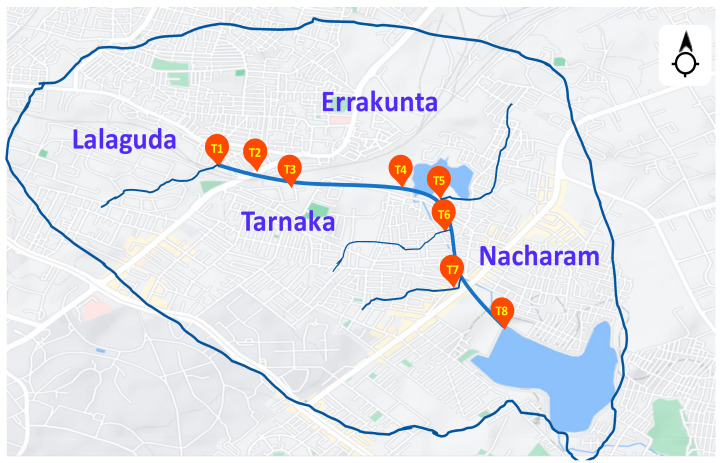
Map showing the point of sample collection (Tarnaka and Nacharam) (sourced from Google Maps).

The study area represents a community with ~2.5 lakhs population covering Tarnaka, HMT Nagar, Lalaguda and Nacharam as part of Greater Hyderabad, Telangana (State), India. The selected community discharges ~30 MLD of domestic wastewater (sewage) which flows through the main drain starting from Lalaguda and finally covering at sampling point T8 before the STP inlet (Figure 1, T8 in our study is considered as the cumulative (composite) representation of all sampling points). Eight sampling points were selected across the drain system to comprehensively represent the majority of lateral drains (Figure 1). Sampling points were selected in such a way as to cover the entire community sewage network. Various lateral drains join the main drain covering the adjoining domestic settlements. Samples were collected at the lateral drain before merging into the main drain. The main sewage drain of the community finally is discharged into the sewage treatment plant (STP) located at Nacharam.

### 2.2. Sampling Details

An optimized sampling protocol was followed to sample the domestic wastewater at the selected points based on our earlier study [22]. The grab samples were collected in a clean plastic bottle (disposable; 1.2 L) containing 20 mL of sodium hypochlorite (0.1%) to inactivate the pathogens. For sample collection, the sample container was placed slightly lowered in the opposite direction of flow with partial immersion. A grab sample volume of one litre was collected at one time with three replicates. Sample information was noted on the field sheets (date and time) along with position (GPS readings), point codes and observations. Grab samples were collected on a weekly and monthly basis. A total of eight samples were collected for weekly monitoring, starting from 7 October 2020 (Week 1), 28 October 2020 (Week 4), 4 November 2020 (Week 5), 11 November 2020 (Week 6) and 18 November 2020 (Week 7). Samples were not collected during Week 2 and Week 3 due to the heavy rainfall events, leading to the overflow of all sewage drains. Monthly samples were sampled at the terminal covering point of the main drain (T8) starting from July 2020, with the intention to long term continous monetering, but due to the monsoon rainfall in early August (14 August 2020 to 24 September 2020 and 10 October 2020 to 21 October 2020), sampling was paused and restarted from October 2020 through August 2021 (Table 1). After sampling, the exposed surface of the container was disinfected (isopropyl alcohol (70%)) and sealed in multi-layered plastic covers, labelled, transported (2–4 °C) to the lab and stored at 4 °C until further processing. Samples were processed within 12 h of sampling for SARS-CoV-2 detection. Biosafety measures were undertaken for sample collection and processing. All the utilities (PPE kit, gloves, cover suite, eye safety glasses, N95 protective mask and shoes) were disposed after use into bio safety bags followed by decontamination. The unused samples and materials were inactivated/disinfected before disposal. Samples were collected between 8:00 and 8:30 am on a day during which there was no rainfall event for 48 h prior to sampling.

### 2.3. Sample Processing

Gravity filtration using 1 mm filter papers was conducted for the collected samples to remove the larger debris, followed by secondary filtration with 0.2 µm filtration units (Nalgene^®^ vacuum filtration system) to remove other fine particles and pathogens [28]. The secondary filtered sample of 60 mL was concentrated to ~600 µL by using 15 mL 30 kDa Amicon^®^ Ultra-15 (Merck Millipore, Burlington, MA, USA) by ultra-filtration (4000 rpm; 4 °C; 10 min). A concentrated 150 μL portion of the sample was used for RNA extraction. All the sample processing and detection experiments were performed in Biosafety level 2 (BSL-2) laboratories.

### 2.4. RNA Extraction and RT-PCR

RNA was extracted from the concentrated samples using the Viral RNA isolation kit (Qiagen, Germantown, MD, USA) as per the provided manufacturer’s protocol. DNA/RNA cross-contamination was avoided by using sterile equipment and RNase-free water for the RNA extraction [22,28]. Isolated SARS-CoV-2 RNA was quantified by using the RT-PCR Detection Kit (Shanghai Fosun Long March Medical Science Co., Ltd., Shanghai, China) which is an FDA (Food and Drug Administration, USA Government) approved kit. Fosun RT-PCR (LoD was 300 copies/mL) contained the primers and chromophore probes encoding for the envelope protein-coding gene (E-gene; ROX), nucleocapsid gene (N-gene; JOE), and open reading frame1ab (ORF1ab; FAM) of SARS-CoV-2. In the RT-PCR assay, reverse transcription (50 °C for 15 min) and the initial denaturation (95 °C for 3 min) were followed by 5 cycles at 95 °C for 5 s and 60 °C for 40 s carried out without data acquisition and 40 cycles with acquisition. Signals from the probes (FAM (ORF1ab), JOE (N gene), ROX (E-gene) and CY5 (Internal reference) were collected by the fluorescence channels at 60 °C [28]. Positive and negative controls of the Fosun RT-PCR kit were also placed for the assay in all the amplifications [28]. C_T_ values in the positive controls matched with given manufactured data and no C_T_ was observed in negative control, which confirmed that the samples were free of cross-contamination. Triplicate analysis was carried out for each sample to obtain more accurate results.

The E-gene, amplified from SARS-CoV-2 RNA, was cloned with KpnI and HindIII restriction sites into a pcDNA3.1 vector and quantified with Qubit™ dsDNA HS Assay Kit (Invitrogen, Carlsbad, CA, USA) and Qubit™ 4 Fluorometer (Invitrogen, Carlsbad, CA, USA). The number of copies per nanogram were calculated based on E-gene and vector sequences were obtained from https://www.ncbi.nlm.nih.gov/nuccore/NC_045512.2?report=fasta&from=26245&to=26472 (accessed on 20 November 2020) and https://www.addgene.org/browse/sequence_vdb/2093/ (accessed on 20 November 2020). The plasmid was diluted from a number of 9.01 log10 to 0.01 log10 copies and RT-PCR was performed. The C_T_ values were plotted against the log copy number and a linear fit equation (R^2^: 0.9993) was obtained [28]. This was used in calculating the number of RNA copies in wastewater based on E-gene C_T_ values. To assess the performance of the qRT-PCR kit used in this work in terms of viral recovery efficiency, 2.14 × 10^7^ pfu/mL viral culture was inactivated at 55 °C for 30 min. RNA was isolated from the heat-inactivated SARS-CoV-2 which was followed by the preparation of log10 dilutions of the RNA. RT-PCR was performed in triplicate for each dilution by following the necessary MIQE guideline. The R2 values obtained from linear regression and efficiency were calculated as described. Detailed data were provided in our previously published study [28]. Inactivated viral culture used in the study was provided to us by Dr. H. H. Krishnan, CSIR-CCMB [28].

### 2.5. Statistical Methods and Data Management

RNA copies/L of wastewater were calculated using the linear fit equation of the E-gene (Equation (1)) [28].

(1) $$Log\;RNA\;copies\;for\;volume\;of\;RNA\;used\;for\;RT-PCR=\frac{C_{T}\;of\;E\;gene-33.696}{-3.2839}$$

The number of infected people in a selected community was calculated by using the two methods in Equations (2) and (3) [12,29].

Method 1 [12]

(2) $$No.of\;infected\;individuals=\frac{\left(\frac{RNAcopies}{Lwater}\right)*\left(L\frac{water}{day}\right)}{\left(\frac{gfaeces}{day}\right)*\left(\frac{RNAcopies}{gfaceces}\right)}$$

Faeces excreted/person/day = 128 g [30] One positive person can shed 10^7^ RNA copies/g of faeces (maximum estimate) [31,32].

Method 2 [29]

(3) $$No.\;of\;infected\;individuals=\frac{\begin{array}{c} No.\;of\;RNAcopiesperliter\\ of\;waste\;water \end{array}}{\begin{array}{c} Contribution\;of\;RNAcopiesper\;person\\ to\;total\;sewage\;water \end{array}}$$

Number of RNA copies/mL of faeces = 10^7^.

Volume of faeces excreted = 120 mL (calculated by considering that the density of human faeces is 1.07 g/mL) [31].

Relative standard deviation (RSD) was calculated for the C_T_ value of individual genes based on Equation (4), where $\bar{X}$ the mean of C_T_ value and *S* is the standard deviation.

(4) $$\mathrm{RSD}\;=100*S/\bar{X}$$

The active phase of infected individuals in the selected sampling area was calculated by considering the total number of infected individuals (based on RNA copies), window period and infection period

(5) $$Individuals\;in\;active\;phase\;of\;infection=\frac{Infected\;individuals\;in\;selected\;area\;}{Window\;period/Infection\;period\;}$$

Window period: 14 days prior to and post sample time (i.e., 29 days in monthly monitoring, including sampling day); Infection period: 14 days (i.e., active symptomatic phase duration).

## 3. Results and Discussion

### 3.1. Weekly Sample Analysis

**Figure 2:**
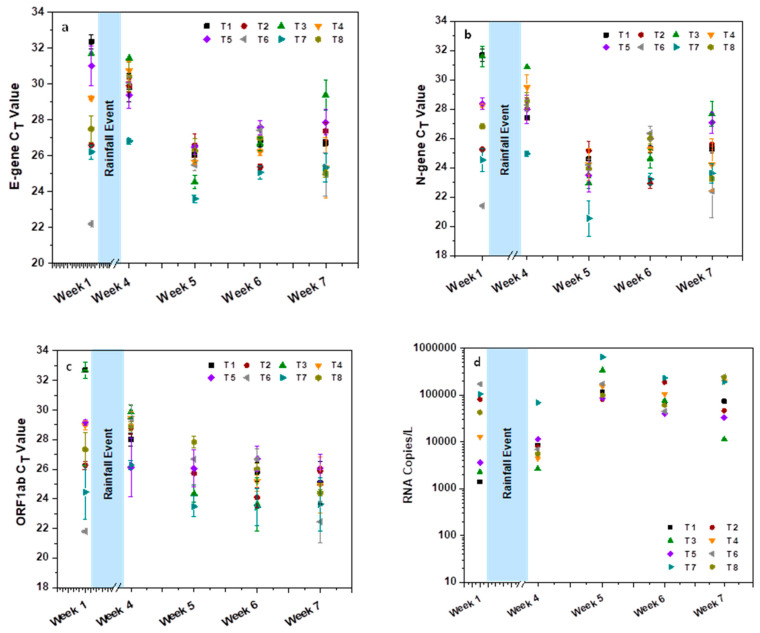
C_T_ values of the (**a**) E-gene, (**b**) N-gene and (**c**) ORF1ab in the wastewater samples collected from various drain discharging domestic sewage in the Tarnaka and Nacharam areas for five weeks, as on 7 October 2020 (week 1) before the Hyderabad floods and continuously for four weeks after the floods on 28 October 2020 (week 4), 4 November 2020 (week 5), 11 November 2020 (week 6) and 18 November 2020 (week 7) during the pandemic; (**d**) RNA copies calculated based on E-gene linear fit equation. The experiments were performed in triplicate.

SARS-CoV-2 genetic material was detected in all 40 samples collected at 8 sampling stations over the window period of 5 weeks from 7 October 2020 to 18 November 2020, with variable loads. Amplification of three SARS-CoV-2 target genes, namely, E-gene, N-gene and ORF1ab, was detected in all the samples (Figure 2). Apart from the C_T_ values, to predict the SARS-CoV-2 viral load in domestic sewage, the RNA copy number was calculated considering the linear fit drawn from the standard curve of the E-gene [28].

At the initial sampling point (South Lalaguda lateral drain; T1), RNA copies of 23,470 copies/L are depicted with the five-week average. Extending the point to the T1, which is the T2 sample (North Lalagudamain drain), showed the RNA copy number of 54,135 copies/L.

An increase in the RNA copy number at the T2 might be due to the convergence of the lateral drains containing domestic sewage discharge into the main drain. Wastewater from the main drain (T2) flows continuously until the end of the selected longitudinal sampling point (Nacharam Inlet to STP; T8). The third sampling point located at Lalapet Bridge (T3) showed a relatively lower incidence of infection dynamics when compared to T1 and T2, with 17,954 RNA copies/L. Even though the main drain stream continued from the earlier sampling point, the number of RNA copies was reported as less at the T3, which might be due to the conflation of dairy processing effluents with excessive surfactants (chemical) discharged into the drain, that may disintegrate the viral RNA material. Similar observations were reported elsewhere [33,34,35]. Downstream from T3, the domestic sewage overflows from Pedda Cheruvu (Lake) and discharges into the main drain (T4). The relative increment in viral load was evident with T4 samples compared to T3, with RNA copies of 45,050 copies/L. In addition to samples collected in the main drain, we have collected samples from lateral drains that were flowing from the set of communities located around the main drain, and this flow finally merged into the main drain. These lateral drain sewage samples depicted marginally lower values of SARS-CoV-2 load than the main drain.

RNA copies of the samples collected at T5 (Errakunta lateral drain) showed 21,757 copies/L. Similarly, the two lateral drains, T6 (VST lateral drain) and T7 (Nacharam lateral drain), presented with higher RNA copies of 119,391 and 184,664 RNA copies/L, respectively. T8 samples representing the terminal point of the main drain where the flow of all the previous sampling points converges showed 51,182 RNA copies/L. C_T_ values of E-gene in all the sampling points ranged between 25.41 ± 1.50% and 28.73 ± 1.04% (Figure 2a). Similarly, the C_T_ values of the N-gene and ORF1ab were observed from 23.37 ± 3.34% to 27.54 ± 1.82% and 19.56 ± 1.33% to 28.72 ± 0.93%, respectively (Figure 2b,c). Three individual genes’ average value of sampling points from T1 to T7 correlated with the C_T_ values observed at the last sampling point, T8. This well-defined correlation indicates the comprehensive epidemiological analysis of the selected community by considering the terminal discharge points of the drain (T8).

#### Community Zoning Based on Infection for Sustainable Management

**Figure 3:**
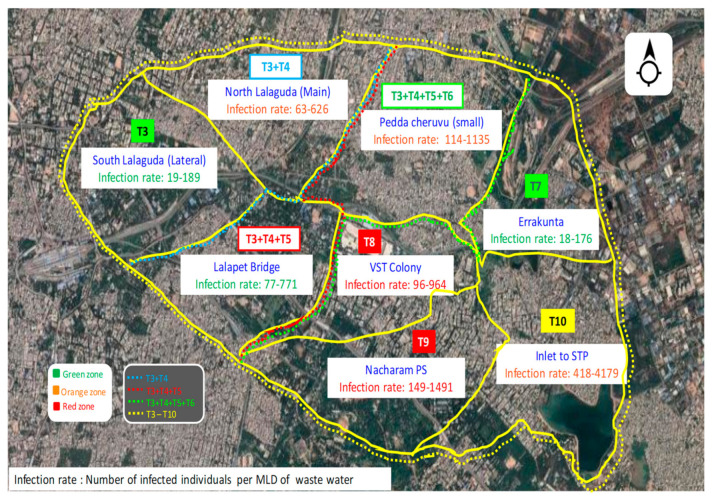
Zoning of the studied community based on the severity of infection (sourced from Google Maps).

Longitudinal and long-term sampling provided the information for the zoning of the selected area based on the severity of the viral spread among the community (Figure 3). As lateral drains were coming from the domestic wastewater outlets, we selected points in such a way that each lateral point represented a defined community based on the discharged sewage. Four major lateral sampling points denote the different distinct zones (T1: Zone I, T5: Zone II, T6: Zone III and T7: Zone IV), whereas the rest of the sampling points were at the main drain that represents the extended zones area in relation to Zone I (T2: Extended Zone I, T3: Second Extended Zone I and T4: Third Extended Zone I). Finally, the terminal sampling point (T8) represents the entire study area, considered as the cumulative major zone. Weekly monitoring to each point over the period of five weeks assisted in studying the spread of the viral genome in the wastewater. The viral genome was detected in all points of sampling in all the weeks, with dynamic changes suggesting the active spread of COVID-19 among the communities. Due to the dynamic variations in the infection rates among the eight sampling points, four zones were divided based on the intensity of infection.

Infection rates were calculated for all the zones, along with cumulative community rates, by considering 10^6^ RNA copies/mL and 10^7^ RNA copies/mL shedding per person (Table 1). For better understanding, we have discussed the infection rates with 10^7^ RNA copies/mL. The number of infected individuals per 1MLDof sewage ranges between 15 and 1500 persons/MLD in all the zones, including distinct and extended. All the zones were categorized into three major classes (Red, Orange and Green) based on the severity of infection. Zones with infection rate <20 persons/MLD were considered as Green zones, where the spread of virus among the community was lower. Zones reporting infection rates >50 persons/MLD were categorized in the Red, representing communities that were more prone to infection with COVID-19. Besides the distinct zones of lateral drains, extended zones of main drain sampling points were also considered for calculating the infection rates where the sewages and populations of two or more distinct zones are jointly represented. Zone I, portraying the south Lalaguda (T1), showed an infection rate of 19 and is considered a Green zone. The T1, T2 sampling point was at the mainstream, so that was considered as the first extended area of Zone I. In the first extension of Zone I, the infection rate was reporting with 63. Though there was a lower infection rate in Zone I, coming to the first extended region the infection rate was significantly increased, thus, this zone was considered Orange (because of joint community and sewage merging). After the T2, sampling points T3 and T4 were in same main drain representing the area of Lalapet and PeddaCheruvu (small). Due to the main stream sewage sampling, these areas were considered as the second and third extended areas of Zone I, with infection rates of 77 and 114. Infection rates in the extended areas consistently increased with the longitudinal increment in the sampling. This is due to the merging of the discharged sewage and join representation of the community. Though there was an infection rate that was >50, these extended zones were considered as the Orange zones, with moderate infection rates. Sampling point T7 (Errakunta) at the lateral sewage drain represented the distinct separate zone (Zone II) where the infection rate (18) was more or less similar to Zone I and is considered a Green zone. Similarly, T6 (VST colony) and T7 (Nacharam) sampling points were lateral sewage drains denoting the two separate independent zones (Zone III and Zone IV). Both the zones were considered as Red zones with infection rates of 96 (Zone III) and 149 (Zone IV). After T7, sampling point T8 (STP inlet) represents the all the zones and is considered as the cumulative major zone, as it is placed nearer to the STP, Nacharam and all lateral and main streams enter into it. Infection rate in the complete major zone (study area) is 418, showing the dynamic and extensive spread of the virus among the population. Among all zones, Nacharam PS areas (Zone IV) were more prone to infection, with a higher number of 149 persons/MLD infection rate, followed by Zone III.

**Figure 4:**
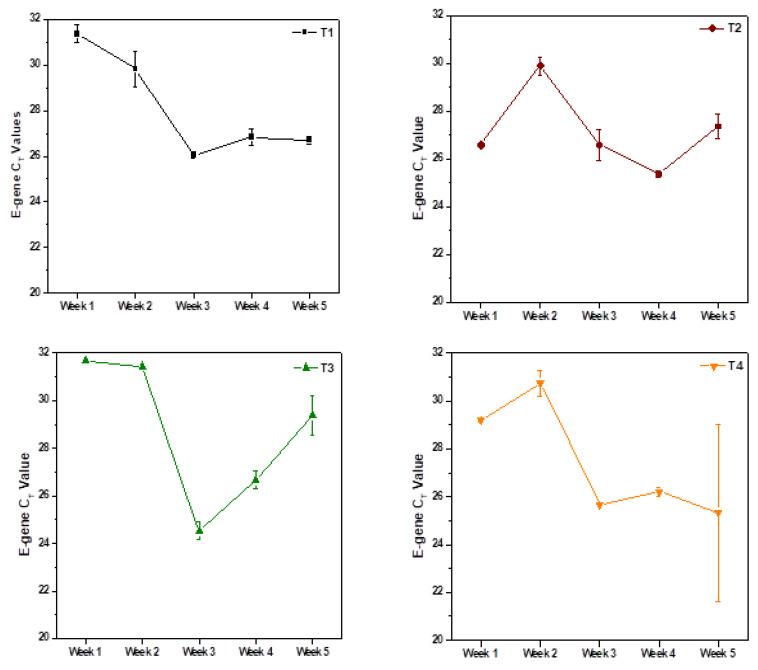

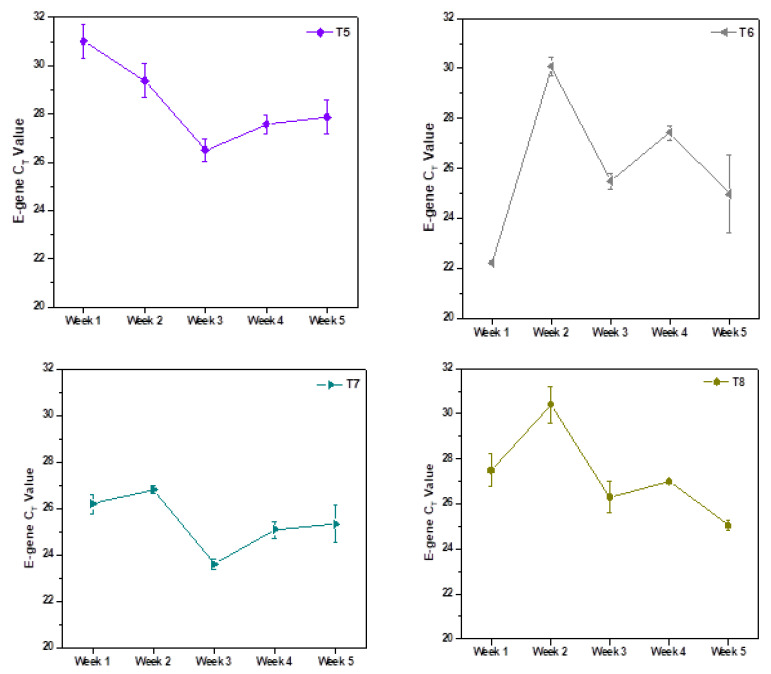
Severity of infection based on E-gene C_T_ values in the sampled point during weekly monitoring (October/November 2020).

In Green zones (T1, T5), E-gene C_T_ values were reported ~28 and above (Figure 4). Particularly, in first two weeks of analysis, C_T_ values were reported as very high, ~31, suggesting the lower spread of the virus, but later on in the third week, higher infections occurred with lower C_T_ values followed by consistent decrease in subsequent weeks. Green zones depicted a similar trend of E-gen C_T_ values over the five weeks of study. Among the eight sampling points, two areas were categorized in the Orange region (T2, T3 and T4) with a greater prone-to-infection rate when compared to the Green regions, with C_T_ of ~27. The C_T_ value profile over the period of five weeks was reported in the dynamic way, with increased and decreased values and vice versa. Dynamics in the C_T_ values of the Orange zones indicate the persistent infection among the community in respect to the extended area of Zone I. Lower C_T_ values <27 were reported in regions of T6 and T7, with these sampling points considered as Red zones with severe infection. Zoning of the selected community helps deliver early warning signals of viral spread in the respective community to tackle and prepare for the pandemic.

### 3.2. Monthly Sample Analysis

**Figure 5:**
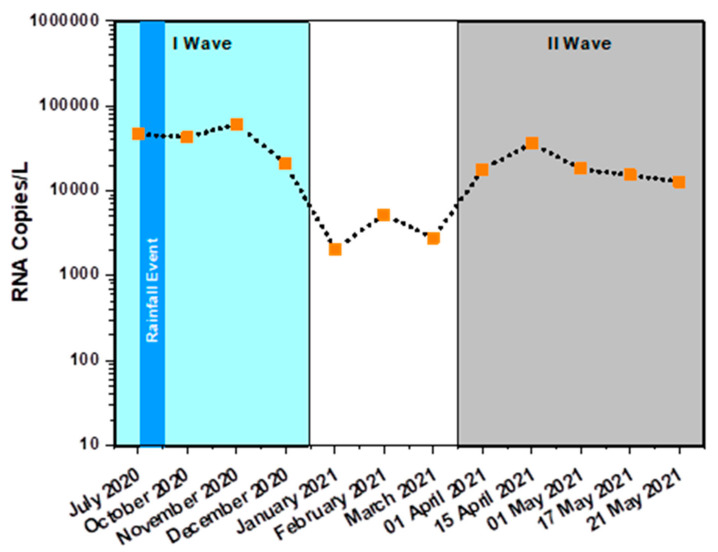
RNA copies calculated based on linear fit equation of E-gene. The experiments were performed in triplicate.

Apart from the weekly monitoring, the dispersive and dynamic viral presence in the domestic sewage was also assessed with long-term (monthly) analysis by selecting the final drain point (T8) as a sampling station, along with a few other stations (T4 to T8). The presence of the three target genes was detected for nine months in one year of monitoring along with samples with variable RNA copy numbers (Figure 5). In July, when the virus was present with 46,527 RNA copies/L after the first month of sampling, multiple rainfall events occurred due to the seasonal monsoon across the Deccan Plateau. Due to the overflow in the drains, sample collections were paused in August and September and were resumed after the flow became normal. In October 2020, 42,772 RNA copies/L were detected, which is more or less similar to July’s viral load analysis data. Specifically, the samples collected during November 2020 showed a higher viral load of 61,160 RNA copies/L with lower E-gene C_T_ values (26.99 ± 0.09%) compared to other monthly samples. This suggests the possibility of high infection rates during November 2020. In December 2020, a considerable drop in the viral load with 20,624 RNA copies/L was recorded, indicating the tapering off of viral load within the community and thus suggesting a decrease in the infection rate. To maintain the accuracy of sampling, from January 2021, samples were collected in five (in some cases three) sampling stations among the eight sampling stations. The viral load during January 2021 reported the lowest infection rates, with 2036 RNA copies/L, and had the highest E-gene C_T_ value at 30.44 ± 0.84% compared to nine months of viral detection. However, compared to January 2021, February 2021 samples showed a marginal increase in viral load with 5228 RNA copies/L, which decreased in the subsequent month of March 2021 with 2781 RNA copies/L. In one year study, a greater number of samples were analyzed during the months of October 2020 and November 2020 because of the weekly sampling and monitoring conducted during that period.

#### Infection Surge and Dynamics

Lowering of the RNA copy number was observed from December through March 2021 (March 2021). After, the March 2021 number of RNA copies increased consistently through the middle of May 2021, suggesting the increase in the viral infection in the community and indicating the second wave period of the viral spread (April: 17,775; Mid-April: 36,399; May: 18,055; Mid-May: 15,301 and End of May: 12,672 RNA copies/L). After the early May 2021 analysis, the number of RNA copies again showed the declining trend and did not detect the viral genome in the June, July and August 2021 samples. This might be due to the dissolution period of the second wave and individual immune development. In the detection period, C_T_ values of E-gene ranged from 26.99 ± 0.09% to 31.84 ± 0.83%, whereas N-gene and ORF1ab ranged between 25.99 ± 0.35% to 30.45 ± 2.22% and 26 ± 0.41% to 28.89 ± 2.70%, respectively (Table 2). Consistent stabilization of RNA copies from April suggested the decline in the infection rates in the upcoming short time period (~15 to 30 days). Temporal variation in the number of infected individuals was observed in our analysis. Such variation might be caused by various factors including infection rate, loss of viral RNA during transit from the source to the sampling site, presence of deteriorating agents in the wastewater samples, and differences in the amount of virus shed by infected individuals. Reports show a loss of 0.02 to 3000 RNA copies/mL during the passage of faecal matter from the point of defecation to the sewage drain [31].

### 3.3. Epidemiological Analysis

Based on the RNA copy number detected in the weekly samples, virus spread in the community was predicted by considering the volume of sewage discharge as well as the population of the selected community (Figure 2 and Table 3). Estimated numbers of infected individuals in the selected community were calculated based on two methods [12,29]. In the present study, 10^7^ RNA copies/mL faeces was used for the description of epidemiological spread [22,28]. The number of infected individuals in the studied community is based on a total window period of 77 days (which includes 14 days each before and after the sampling period combined with 49 days of the sampling period). The window period was selected based on the reports that showed the persistence of SARS-CoV-2 genetic material in the faecal matter of infected individuals before, during and after the active infection phase [14,36]. The probable number of infected individuals in the studied community (77 days window period) was 1567, with 285 people being in the active phase of infection (Table 3). The community under study covers a population of ~2.5 lakhs, which represents ~1.79% of the total Hyderabad city population, considering the number of infected individuals in the selected community with 30 MLD domestic sewage flow (i.e., 1.6% of total sewage flow of 1800 MLD of Hyderabad city) [37]. An extrapolation was performed to arrive at the total number of infected individuals of the city, which was calculated to be 94,018, with about 17,094 active phase individuals. The infection rate of 52 persons/MLD was derived based on infected individuals and population figures (Table 3).

Using the monthly monitoring data, the number of infected individuals was calculated during each month individually (Table 4). The number of infected individuals in the month of July 2020 was recorded as 1127 (study area) and 67,610 (total city), with active phase individuals of 537 and 32,195. After the rainfall event during the months of August and September, the number of infected individuals reported in October 2020 was more or less similar to that in July 2020, wherein infected/active phase individuals in the study area and the entire city were 1036/493 and 62,153/29,597, respectively. Marginal variation from July to October indicates the consistent spread of the viral load among the community. However, in November 2020, the infection rates rose significantly (infected/active, 1481/705 (study area); 88,873/42,321 (city)). A substantial increase in the number of infected individuals in November 2020 suggests widespread SARS-CoV-2 infection in the community. In subsequent months, December 2020 showed a substantial drop in infection (499/238 study area); (29,969/14,271 city). This decrease was observed in January 2021, with a minimal number of infections ((infected/active, 49/23 (study area); 2959/1409 (Hyderabad)) when compared to the nine months of viral detection in the one-year study. However, February 2021 data indicated a minor increase in infected individuals of 127 and 7597 in the study area and Hyderabad city, respectively, having an active phase individual count of 60 (selected community) and 3618 (Hyderabad) compared to January 2021 and followed by a decrease in March 2021 (67/32 study area; 4041/1924 in city).

As RNA copies increased from March 2021 onward, the number of infected individuals also increased substantially through the first half of the May 2021 and decreased by the end of May ((April: infected/active 430/205 study area; 25,829/12,300 in Hyderabad); (Mid-April: infected/active 882/420 study area; 52,892/25,187 in Hyderabad); (May: infected/active 437/208 study area; 26,236/12,493 in Hyderabad)) These increases might suggest a second term spread of the virus. After, the mid-May 2021 number of infected individuals again attained the consistent declined state; this might be due to the dissolution wave spread of the virus. Samples collected in June 2021, July 2021 and August 2021 showed no viral targeted genes. A higher number of infected individuals were reported in the month of November 2020 and the lowest infection was recorded in January 2021 and March 2021. The infection rate showed more or less similar load from July to October, with the incremental trend in November 2020 followed by a wave phase trend with a decrease (December 2020 to March 2021) to increase (March 2021 to May 2021) for subsequent months of analysis. The repeated detection of the viral RNA over the months in sewage indicates infection severity and persistence of SARS-CoV-2. It is evident from sewage surveillance data that 64% of the Hyderabad population was infected with SARS-CoV-2, which was close resembles the Sero-analysis data performed by CSIR-CCMB, which reported infection rates to be 54% [38].

The WBE tool is one of strategies used to track the disease dynamics of viral infections in a given community with minimal samples. Longitudinal sampling from the different selected points provides an appropriate state of viral infection within the selected community [39,40]. The number of infected individuals reported in the present study includes pre- and post-symptomatic, asymptomatic and mildly symptomatic individuals. Associated with clinical data, WBE could provide critical monitoring of SARS-CoV-2 transmission within a community including the beginning, tapering off or reemergence of the virus [41,42,43,44,45].

The WBE studies of infectious pathogens offer unbiased monitoring of infection prevalence, spreading rate and dynamics of infection in terms of special and temporal avenues. Previously, we estimated the percentage of infected population in Hyderabad based on the sewage viral load detected in various sewage treatment plants (STPs) across the city. In view of the fast spreading ability of SARS-CoV-2 over time, it is mandatory to establish the potential surveillance approach to the community level for better management of the infection. In this work, we presented the long-term SARS-CoV-2 sewage surveillance by longitudinal sampling in a selected community. As previously described, we calculated the number of infected individuals based on the viral RNA copies present in sewage water. The viral load in the samples at different time points clearly indicated the infection dynamics during the study time of one year. Our calculation showed the maximum number of infected individuals during the month of November, which is in agreement with the clinical diagnostic data during the same period of time. This observation clearly supports the advantage of sewage surveillance at the community level, which could help in providing better clinical care.

In addition, we noticed a pattern in the viral load in sewage samples. It reached a saturation or steady state at some point during the time course and gradually decreased later. The calculated percentage of the infected population from long-term surveillance not only aids in understanding the disease dynamics but also provides an opportunity to predict the establishment of herd immunity among the population. This can be achieved by calculating the number of infected individuals’ overtime with respect to the total population. Since the estimations are purely based on the sewage viral load; the sampling conditions play crucial roles. To achieve clear and reliable estimations, it is important to observe the viral load trends for more than onetime point. If there is a sudden rise in the viral load, repeated periodical sampling and assessment would help in reaching better and more comprehensive conclusions. For long term surveillance, sampling and processing shall be performed once or twice every month from different communities or areas. The occurrence of SARS-CoV in sewage originating from the hospital during the SARS-CoV-1 outbreak was tested for its virulence/occurrence before and after disinfection using a symptomatic patient sample [46]. Genomic material of the virus could be detected by targeting the specific coding genes through the RT-PCR. The detection of the virus in sewage when the SARS-CoV-2 prevalence was low indicated the functional role of sewage for surveillance to monitor the circulation of the virus in the population via wastewater-based epidemiology (WBE) [28,47]. The surveillance/monitoring of SARS-CoV-2 in wastewater could be able to quantify the scale of infection prevailing among the community, with the benefit of detecting the virus from symptomatic, asymptomatic or pre-symptomatic cases which manifest as an early-warning sign (Table 5). The WBE approach will help to minimize the outbreak spread and also serve for future epidemic surveillance.

## 4. Conclusions

The spread and persistence of SARS-CoV-2 infection was studied by detecting the viral genetic material in the domestic sewage using the longitudinal grab sampling protocol in a selected community through a long-term epidemiological surveillance. Sample collection from the lateral drains along with the mainstream provided information about the dynamics of infection among the community. The detection of viral genetic material in the sewage over the period of nine months indicates the persistence of SARS-CoV-2 among the studied community. Considering the capacity of sewage generation, we have calculated the number of infected peoples along with the active infectious phase. Based on the long-term representative sampling, we have tried to extrapolate data to the total city to understand the dynamics of the outbreak of the virus. Continuous monitoring over the period of nine months, including weekly monitoring, helped to understand the viral load in the selected community based on the discharged domestic wastewater and assisted in determining the infection status of the particular community as well as zoning of the community based on the infection rate. This WBE study offers an early warning system as well as provides a clear view of infection dynamics and immunity status of the population, as asymptomatic, symptomatic and pre-symptomatic individuals shed virus. Moreover, these findings are unbiased. Performing WBE studies can be extended to the surveillance of other infectious pathogens, as the method is simple to perform yet efficient enough to help understand the infection type and dynamics among the population in a temporal manner.

## Figures and Tables

**Table 1:** Weekly and monthly sampling details with respect to the longitudinal location.

Weekly Monitoring					
S. No.	Sampling Point	Location	Drain	Sample Code	Sampling Date and Time
1	South Lalaguda (Point–3)	South Lalaguda	Lateral Drain	T1	7 October 2020 28 October 2020 4 November 2020 11 November 2020 18 November 2020 8:00 to 8:30 am
2	North Lalaguda (Point–4)	North Lalaguda	Main Drain	T2	
3	Lalapet	Lalapet Bridge	Main Drain	T3	
4	Tarnaka (Drain–1)	Near PeddaCheruvu (Small)	Main Drain	T4	
5	Tarnaka (Drain–2	Errakunta	Lateral Drain	T5	
6	Tarnaka (Drain–3)	VST Colony	Lateral Drain	T6	
7	Tarnaka (Drain–4)	Behind Nacharam PS	Lateral Drain	T7	
8	Nacharam (Drain–1)	Inlet to STP	Main Drain	T8	
**Monthly Monitoring**					
		Location; Point of Drain		Sampling date (8:00 to 8:30 am)	
1	Nacharam	T8		22 July 2020	
2	All (eight) sampling points	T1 to T8		7 October 2020 4 November 2020	
	Nacharam	T8		11 December 2020	
3	Five sampling points	T4–T8		20 January 2021 13 February 2021 2 March 2021	
4	Three sampling points	T4, T7 and T8		1 April 2021 15 April 2021	
5	Three sampling points	T4, T7 and T8		1 May 2021 17 May 2021 21 May 2021	
6	Three sampling points	T4, T7 and T8		4 June 2021 18 June 2021 24 June 2021	
7	Three sampling points	T4, T7 and T8		4 July 2021 10 July 2021 14 July 2021 27 July 2021	
8	Three sampling points	T4, T7 and T8		4 July 2021	
**Zoning of Study Area**					
	**Zones**	**Covering Areas**		**Infection Rate**	
1	Zone I	T1		19–189	
	(i) Extended Zone I	T1+T2		63–626	
	(ii) Second Extended Zone I	T1+T2+T3		77–771	
	(iii) Third Extended Zone I	T1+T2+T3+T4		114–1135	
2	Zone II	T5		18–176	
3	Zone III	T6		96–964	
4	Zone IV	T7		149–1491	
5	Cumulative Major Zone	T1 to T8 (Complete study area)		418–4179	

**Table 2:** SARS-CoV-2 RNA load with monthly monitoring samples.

Sample Code	E-Gene * (C_T_)		N-Gene * (C_T_)		ORF1ab * (C_T_)		RNA Copies/L **
	T8	Average of Longitudinal Sampling	T8	Average of Longitudinal Sampling	T8	Average of Longitudinal Sampling	
July 2020	27.38 ± 0.36%	SC	26.12 ± 1.38%		28.02 ± 1.92%		46,527
October 2020	27.5 ± 2.65%	28.34 ± 1.25	26.82 ± 0.63%	27.24 ± 1.25	27.34 ± 4.10%	27.92 ± 2.09	42,772
November 2020	26.99 ± 0.09%	26.49 ± 0.96	25.99 ± 0.35%	25.19 ± 1.65	26 ± 0.41%	25.37 ± 3.23	61,160
December 2020	28.5 ± 0.21%	SC	26.84 ± 0.73%	SC	27.19 ± 0.49%	SC	20,624
January 2021	31.84 ± 0.83%	30.44 ± 0.84%	29.49 ± 4.26%	29.48 ± 4.81%	28.89 ± 2.70%	28.99 ± 2.15%	2036
February 2021	30.50 ± 1.30%	31.10 ± 1.98%	30.45 ± 2.22%	31.23 ± 2.95%	28.30 ± 5.48%	28.77 ± 2.33%	5228
March 2021	31.39 ± 2.86%	31.38 ± 1.88%	29.38 ± 1.30%	29.53 ± 1.11%	27.63 ± 3.38%	27.12 ± 3.35%	2781
1 April 2021	29.28 ± 2.27%	30.08 ± 1.99%	28.09 ± 2.20%	26.12 ± 0.93%	26.71 ± 2.61%	27.43 ± 2.91%	17,775
15 April 2021	27.73 ± 0.52%	27.54 ± 1.04%	26.12 ± 0.93%	25.91 ± 1.57%	27.59 ± 0.98%	27.21 ± 1.89%	36,399
1 May 2021	28.73 ± 0.63%	28.63 ± 1.12%	27.46 ± 0.56%	27.24 ± 1.23%	28.06 ± 0.26%	27.23 ± 3.21%	18,055
17 May 2021	28.97 ± 0.18%	29.01 ± 0.67%	26.03 ± 0.48%	28.97 ± 0.18%	26.77 ± 0.15%	26.56 ± 0.56%	15,301
21 May 2021	29.23 ± 1.44%	28.78 ± 1.04%	26.27 ± 1.57%	25.51 ± 2.60%	25.01 ± 1.36%	25.42 ± 2.24%	12,672
4 June 2021	Below the detectable limits						
18 June 2021							
24 June 2021							
4 July 2021							
10 July 2021							
14 July 2021							
27 July 2021							
8 August 2021							

* Represent $\bar{X}$ +RSD; ** RNA copies (based on E-gene) were calculated based on the linear fit equation. SC—Samples were not collected.

**Table 3:** Infected individuals estimated during the sampling window, which includes symptomatic, asymptomatic and recovered, of weekly monitored samples.

Sample	Capacity of the STP (in MLD)	RNA Copies/Person Contribution to STP (10^7^ Copies/mL Faeces)	Method 1	Method 2	RNA Copies/Person Contribution to STP (10^6^ Copies/mL Faeces)	Method 1	Method 2
T1	30	40	550	587	4	5501	5868
T2			1269	1353		12,688	13,534
T3			421	449		4208	4489
T4			1056	1126		10,559	11,263
T5			510	544		5099	5439
T6			2798	2985		27,982	29,848
T7			4328	4617		43281	46,166
T8			1200	1280		11,996	12,796
Infected individuals (for study area with 30 MLD; ~2.5 Lakh)			1516	1618		15,164	16,175
Average estimate of infected individuals (for study area with 30 MLD; ~2.5 Lakh)				1567			15,670
Estimate of the population in active phase of the infection during the window period of 77 days (for study area with 30 MLD; ~2.5 Lakh)				285			2849
Infected individuals for 1800 MLD (on total sewage generation of Hyderabad city)			90,985	97,051		909,849	970,506
Average estimate of infected individuals for 1800 MLD (on total sewage generation of Hyderabad city)				94,018			940,177
Estimate of the population in active phase of the infection during the window period of 77 days for 1800 MLD (on total sewage generation of Hyderabad city)				17,094			170,941

**Table 4:** Disease dynamics and infection rate estimated during the sampling window of nine months, which includes symptomatic, asymptomatic and recovered.

Sampling Month	Average Estimate of Infected Individuals (in Study Area of 30 MLD)		Estimate of the Individuals in Active Phase of the Infection during the Window Period (29 Days)		Estimate of Infected Individuals of Hyderabad City (1800 MLD)		Estimate of the Individuals in Active Phase of the Infection during the Window Period (29 Days)		Infection Rate (Person/MLD of Sewage)	
	10^7^ Copies/mL Feces	10^6^ Copies/mL Feces	10^7^ Copies/mL Feces	10^6^ Copies/mL Feces	10^7^ Copies/mL Feces	10^6^ Copies/mL Feces	10^7^ Copies/mL Feces	10^6^ Copies/mL Feces	10^7^ Copies/mL Feces	10^6^ Copies/mL Feces
July 2020	1127	11,268	537	5366	67,610	676,095	32,195	32,1950	38	376
October 2020	1036	10,359	493	4933	62,153	621,531	29,597	295,967	35	345
November 2020	1481	14,812	705	7053	88,873	888,731	42,321	423,205	49	494
December 2020	499	4995	238	2379	29,969	299,693	14,271	142,711	17	166
January 2021	49	493	23	235	2959	29,586	1409	14,088	2	16
February 2021	127	1266	60	603	7597	75,969	3618	36,176	4	42
March 2021	67	674	32	321	4041	40,411	1924	19,244	2	22
1 April 2021	430	4305	205	2050	25,829	258,293	12,300	122,997	14	143
15 April 2021	882	8815	420	4198	52,892	528,923	25,187	251,868	29	294
1 May 2021	437	4373	208	2082	26,236	262,362	12,493	124,934	15	146
17 May 2021	371	3706	176	1765	22,234	222,343	10,588	105,877	12	123
21 May 2021	307	3069	146	1461	18,414	184,140	8769	87,686	10	102
4 June 2021	Not calculated because of low detection limits									
18 June 2021										
24 June 2021										
4 July 2021										

**Table 5:** Various sampling methods adopted and detection assay for estimation of virus in domestic wastewater.

Virus	Sampling	Detection by RT-PCR Assay/Target Gene Used	Reference
SARS-CoV-1	Grab sampling: Sewage wastewater from two SARS patients	RT-qPCR/Three sets of primers to detect the SARS-CoV RNA: Cor-p-F2, Cor-p-F3 and Cor-p-R1	[46]
Coronaviridae (Virome); Alphacoronavirus; Betacoronavirus	Grab sampling; Water body	Nuclic acid library preparation for sequencing to compare viromes	[47]
SARS-CoV-2	Grab and composite sampling; Sewage wastewater	ORF1ab, E, N and S	[48]
	Grab and composite sampling; Untreated doemsic wastewater	RT-qPCR;N_Sarbeco and NIID_2019-nCOV_N	[12,13]
	Composite sampling; Untreated sewage wastewater.	CDC N2	[49]
	Grab Sampling; Inlets and outlet of WWTP/STP and septic tank influent of hospital	CCDC-ORF1 andCCDC-N	[50]
	Composite sampling; Untreated wastewater	NA	[51]
	Composite sampling; Untreated and treated wastewater	M and RdRP	[35]
	Grab and composite sampling; Untreated wastewater	ORF1ab, N and E	[52]
	Grab sampling; Influent and secondary treated wastewater	N_Sarbeco NIID_2019-nCOV_N CDC N1, N2 and N3	[6,53]
	Grab samples, Influent; Secondary and tertiary effluents	CDC N1, N2, N3	[6]
	Grabs and composite sampling; Secondary treated effluent, untreated and treated wastewater	CDC N1, CDC N2 and CDC N3	[54,55,56]
	Grab samples; inlet and outlet of STP/WWTP	E, N and ORF1ab	[28]
	Grab and composite sampling; Drains	E, N and ORF1ab	[22]
	Grab sampling; Drains	RT-PCR; N1, N2 and N3	[57]
	Passive sampling device: Sewage wastewater	N2	[58]
	Grab and composite sampling; WWTP	N1 and N2	[59]
	Grabsampling; Drains	ORF1ab	[60]
	Composite sampling (Auto-sampler); Inlet of WWTP	E, RdRp and N	[61]
	Composite sampling; WWTP/STP	N	[62]
	Composite sampling; WWTP	N, S and ORF1ab	[63]
	Grab Sampling; Water bodies	E, N and ORF1ab	[64]

NA—Information not provided.

## Data Availability

Not applicable.
